# Organic carbon cycling and black shale deposition: an Earth System Science perspective

**DOI:** 10.1093/nsr/nwad243

**Published:** 2023-09-15

**Authors:** Zhijun Jin, Xiaomei Wang, Huajian Wang, Yuntao Ye, Shuichang Zhang

**Affiliations:** Institute of Energy, Peking University, Beijing100871, China; Key Laboratory of Petroleum Geochemistry, Central Laboratory of Geological Sciences, Research Institute of Petroleum Exploration and Development, China National Petroleum Corporation, Beijing100083, China; Key Laboratory of Petroleum Geochemistry, Central Laboratory of Geological Sciences, Research Institute of Petroleum Exploration and Development, China National Petroleum Corporation, Beijing100083, China; Key Laboratory of Petroleum Geochemistry, Central Laboratory of Geological Sciences, Research Institute of Petroleum Exploration and Development, China National Petroleum Corporation, Beijing100083, China; Key Laboratory of Orogenic Belts and Crustal Evolution, Ministry of Education, School of Earth and Space Sciences, Peking University, Beijing100871, China; Key Laboratory of Petroleum Geochemistry, Central Laboratory of Geological Sciences, Research Institute of Petroleum Exploration and Development, China National Petroleum Corporation, Beijing100083, China

**Keywords:** black shale, Earth System Science, carbon cycle, petroleum resource, climate change

## Abstract

Earth has a prolonged history characterized by substantial cycling of matter and energy between multiple spheres. The production of organic carbon can be traced back to as early as ∼4.0 Ga, but the frequency and scale of organic-rich shales have varied markedly over geological time. In this paper, we discuss the organic carbon cycle and the development of black shale from the perspective of Earth System Science. We propose that black shale depositions are the results of interactions among lithospheric evolution, orbital forcing, weathering, photosynthesis and degradation. Black shales can record Earth's oxygenation process, provide petroleum and metallic mineral resources and reveal information about the driver, direction and magnitude of climate change. Future research on black shales should be expanded to encompass a more extensive and more multidimensional perspective.

## Teaser

Black shale depositions are valuable archives of interactions between different geological forcings.

## INTRODUCTION

**Figure 1.**
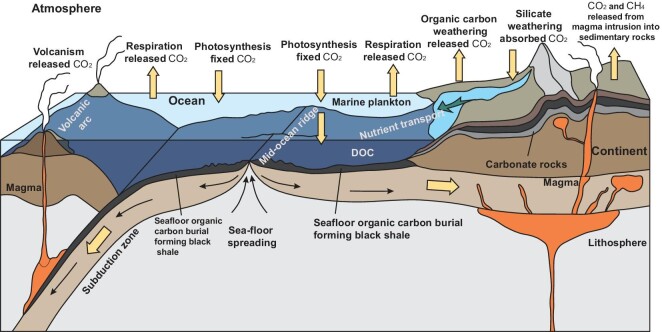
Organic carbon cycle and black shale development driven by interactions of Earth’s spheres. DOC, dissolved organic carbon. Modified from [1,2].

Earth's surface can be divided into four ‘spheres’: the lithosphere, biosphere, atmosphere and hydrosphere. The cycling of organic carbon produced by organisms in the atmosphere, oceans, land and mantle reservoirs is a particular characteristic of Earth [1,2] (Fig. 1). The ‘Gaia hypothesis’ proposed by Lovelock suggests that the Earth system—a synergistic complex entity—ensures the evolution of Earth as a planet sustaining the development of life [3]. In this process, the advantage of biological oxygenic photosynthesis over respiration has led to the accumulation of organic carbon on Earth and the oxygenation of the atmospheric and marine environments [4].

The lithosphere is one of the most significant reservoirs of organic carbon [1]. It was estimated that the amount of organic carbon fixed in Earth's crust may be as high as 8100–15 600 × 10^12^ t [4]. Strikingly, organic-rich mudstones or siltstones with well-developed laminations are often referred to as ‘black shales’. Although their colors can range from dark gray to black, rocks containing abundant organic materials at low maturity are commonly brown, especially after weathering. A threshold of the total organic carbon (TOC) content for black shales has not been well defined because TOC can decreases with thermal evolution and hydrocarbon production. Based on empirical investigations, black shales with TOC contents of >0.5 wt% are commonly regarded as effective hydrocarbon source rocks in the petroleum industry [5,6].

The ocean is another important reservoir of organic carbon on Earth's surface environment, which is held mainly in the form of dissolved organic carbon (DOC) that is associated with activities of marine microorganisms [7]. The size of the DOC reservoir in the modern ocean might be ≤680 Gt, which is comparable to the atmospheric carbon dioxide (CO_2_) reservoir (720 Gt) [8]. On the basis of geological modeling and carbon burial flux calculations, the magnitude of the marine DOC reservoir on early Earth was suggested to be hundreds to thousands of times larger than that in the modern ocean [9,10]. The geological lifetime of oceanic DOC may be in the order of millennia (kyr) to millions of years (Myr) [1,7] but this form of carbon is unstable and may have substantial impact on the atmospheric oxygen (O_2_) and CO_2_ reservoirs. Moreover, it may have considerable implications for global climate change and the sedimentation of black shale.

Pioneer geologists used black shales as marker layers for stratigraphic correlation and have made outstanding achievements in mineralogy, petrology, paleontology and many other fields. With the discovery of hydrocarbon resources, the research of black shale has received great attention. For instance, studies of Mesozoic lacustrine shales laid the foundation for the theory of continental petroleum geology [11]. In recent years, advances in Earth System Science have required scientists to reconceptualize black shale deposition with respect to a more integrated spatial and temporal context. It has also been realized that black shale is a product of matter and energy exchange between different spheres of Earth. Accordingly, this paper considers black shale as an information and resource carrier regarding the evolution of Earth, focusing on the interactions among various spheres of the Earth system and their impacts on human social and economic development in order to better understand the past, present and future of Earth.

## BLACK SHALE IN GEOLOGICAL HISTORY

**Figure 2.**
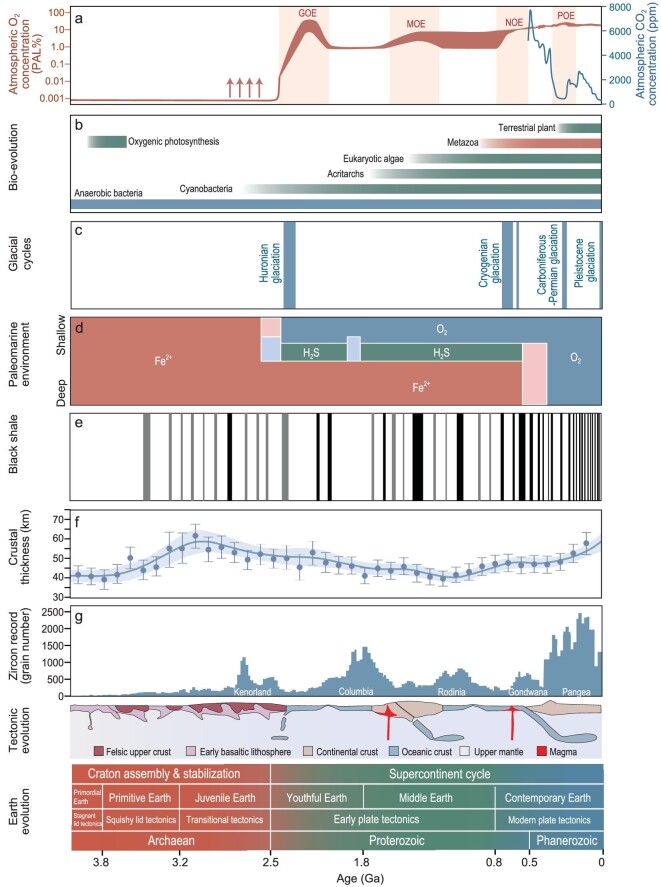
Earth's evolution and black shale development. (a) Atmospheric O_2_ [14,15] and CO_2_ levels [16]. (b) The time scale of life evolution. (c) Major glaciations over geological history. The Cryogenian glaciations include the older Sturtian and the younger Marinoan epochs. (d) Oceanic redox states. Pink and light blue denote the transitions between ferruginous (Fe^2+^) and oxic waters (O_2_) and sulfidic waters (H_2_S), respectively [17]. (e) Stratigraphic distribution of black shales, summarized from [6,18]. Black barcode represents global occurrence of black shale, while gray interval indicates local development. (f) Crustal thickness, adopted from [19]. (g) Zircon records as well as the stages of tectonic evolution are from [20].

Earth formed ∼4.6 billion years ago. The oldest known organic matter is in iron-bearing sedimentary rocks of the Nuvvuagittuq supracrustal belt, Canada (4.28–3.77 Ga) and in metamorphic rocks of the Isua supracrustal belt, west Greenland (∼3.78 Ga) [12]. The oldest black shales with depositional ages of 3.5–3.2 Ga occur in ancient cratons, such as the Pilbara in western Australia and the Kaapvaal in South Africa [13]. The development of black shales after this time started to show a global distribution featured by high spatio-temporal continuity and diversity (Fig. 2) [6,14–20]. Ten sets of black shales were developed in the Pilbara Craton from 3.0 to 2.45 Ga [21]. Shales dated at ∼2.71 Ga were deposited in the Pilbara and Kaapvaal cratons, the Abitibi greenstone belt in Canada and the Carajás Basin in Brazil, with TOCs of ≤29.9 wt% [21]. Black shales of the Great Oxygenation Event (GOE; 2.2–1.8 Ga) were developed in all major cratons worldwide. In particular, the positive carbon isotope excursions of Lomagundi at 2.22–2.10 Ga are globally isochronous [22,23]. Black shales developed during 1.8–1.3 Ga were preserved mainly in North China, northern Australia, India, Siberia and Baltica [6]. Hydrocarbon source rocks with ages of 1.3–0.8 Ga were exposed in regions of the Shennongjia, Laurentia, Siberia, Baltica, India, West Africa and São Francisco [6].

Since 0.8 Ga, the records of black shales have become more widely distributed, exhibiting a notable increase in TOC contents compared with their older counterparts (Fig. 2). These younger black shales contribute to >99% of known hydrocarbon resources. During the Jurassic and Cretaceous, frequent global oceanic anoxic events occurred, which were conducive to black shale deposition. These events resulted in widespread black shale depositions hosting >56% of current hydrocarbon resources [24]. One potential explanation for this phenomenon is that the younger shales are better protected by overlying sediments. In contrast, Precambrian black shales with much longer geological history might have undergone recycling [20].

## REGULATION OF ORGANIC CARBON AND BLACK SHALE DEVELOPMENT BY MULTI-SPHERE INTERACTIONS

The formation and preservation of black shale are the result of interplays between biological activity, marine environment and sedimentary evolution. The traditional theory emphasizes the primary productivity and preservation condition in models of black shale formation [5]. However, Earth System Science concerns not only spatial reorganization and energy exchange, but also the interaction of different processes over various temporal scales. Although black shales have formed throughout most of geological history, they are distinctly inhomogeneous, indicating that multiple geological interactions should be considered regarding black shale depositions.

### Organic carbon cycling driven by tectonic processes

Rather than a monolithic layer, the present-day lithosphere is a patchwork of tectonic plates. Plate activity is a typical characteristic that differentiates Earth from other planets and the margins of these plates are the most important channels for material and energy circulation between Earth's interior and surface [2]. Magmatic activity in Earth's interior not only exerts strong influences on the distribution of lands and oceans, but also affects global climate through physicochemical processes such as greenhouse gas release and rock weathering. The increasing frequency of black shale deposition through time as documented in the geological record is consistent with increasing plate activity, suggesting that black shale deposition might have been driven by lithospheric evolution and associated tectonic forcings (Fig. 2).

Earth's continental crust probably formed between 3.8 and 3.0 Ga. The oldest evidence of subduction suggests the initiation time of plate tectonic activity at ∼3.0–2.5 Ga, but plate subduction systems covering most of the globe may not have started until ∼2.0 Ga [25]. Prior to the initiation of plate movement, the input of nutrients required for biological activity might have been limited. Volcanic activities and submarine hydrothermal fluids were critical for the supply of key nutrients in the ocean and for the maintenance of biological activity at that time, which in turn controlled the formation and number of black shales [13]. The first phase of crustal thickening occurred between 3.8 and 3.2 Ga [26], forming long-term undeformed and stable continents during the process known as cratonization. Cratons, as rigid blocks, overlie the asthenosphere and are able to maintain stability during later geological times, thus providing long-term protection for black shale preservation.

**Figure 3.**
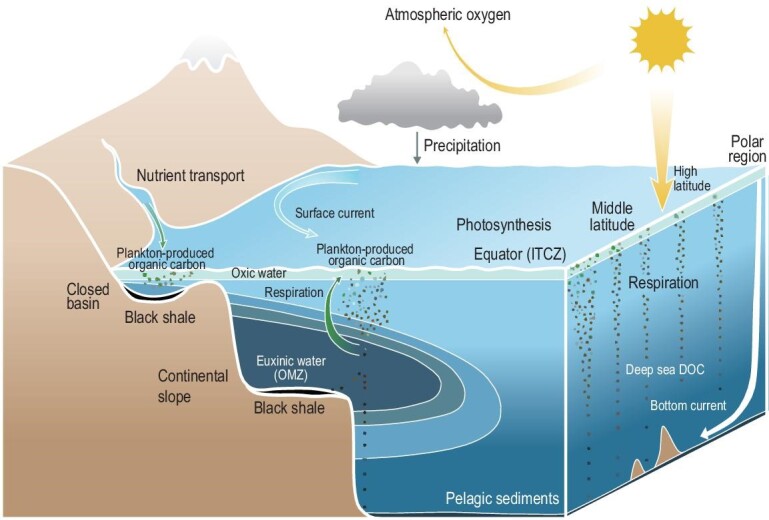
Nutrient transport through weathering leads to increased marine primary productivity, anoxic water columns and black shale development. Light blue indicates a higher concentration of dissolved oxygen, while dark blue indicates a lower oxygen level. ITCZ, Intertropical Convergence Zone; DOC, dissolved organic carbon; OMZ, oxygen minimum zone.

The Paleoproterozoic Era as a period of major transition during Earth's evolution witnessed the formation of the first supercontinent or supercraton (Kenorland) [27]. The activity of large igneous provinces (LIPs) on the stable supercraton resulted in extensive development of rifted basins [20]. Volcanic activity could have enhanced terrestrial weathering through the accumulation of CO_2_, delivered large amounts of nutrients to the ocean and provided a favorable environment for photosynthetic organisms to flourish, which could cause pronounced sequestration of CO_2_ and a considerable increase in oxygen production [28,29]. Atmospheric oxygenation also enhanced terrestrial aerobic weathering, supplying more soluble sulfates and key metal nutrients, such as Mo and Cu, to the ocean [22,23]. This process resulted in a burst of primary productivity and the formation of anoxic sulfidic waters, which in turn facilitated the burial of organic carbon and sustained the release of oxygen (Fig. 3), eventually inducing positive feedback between atmospheric oxidation and black shale development. Therefore, the GOE was the first ‘redox event’ over geological history.

The evolution of Earth's lithosphere during 1.8–0.8 Ga was characterized by crustal thinning [19,20]. The lack of clear evidence for rifting and reorganization of major cratons (e.g. Laurentia, Siberia and Baltica) supported the view of a relatively continuous evolution from supercontinent Columbia to Rodinia (Fig. 2) [19]. This supercontinent might have exerted a thermal overburden effect on the mantle, leading to warming and weakening of the continental lithosphere, limiting both the height of mountains that can be mechanically supported and nutrient flux into the ocean [19]. Such a long-term stable environment significantly affected the formation of black shale. A typical feature of hydrocarbon source rocks during this period is that it was associated mostly with global LIP events or local volcanic activity [30], implying that magmatic activity was critical for black shale formation during periods of crustal thinning and orogenic quiescence [31]. Notably, LIP activities could have changed the long-term environmental stasis and provided the necessary nutrients for black shale development. In general, terrestrial LIPs are known to have life spans of <1 Myr, although some lasted as long as 5 Myr [32]. However, geochronology of black shales shows that these shales were often deposited for periods of >1 Myr and some Precambrian black shales may have been deposited for >30 Myr, such as the Xiamaling Formation at 1.4 Ga and the time-equivalent Velkerri Formation [33]. Therefore, major geological events, such as magmatism, are more likely drivers that may have triggered feedback loops to sustain longer-term deposition of black shales.

Many typical features of Phanerozoic plate tectonics were already evident during the Neoproterozoic Era (1.0–0.54 Ga), including the spreading of mid-oceanic ridges, plate margin subduction and collisional and accretionary orogenesis [20]. Earth's climate and biosphere have also undergone abrupt changes since 0.8 Ga. It is a period characterized by frequent global glaciations and a significant increase in Earth's surface oxygenation levels [28]. Eukaryotic algae, although they appeared relatively early at the terminal of the Paleoproterozoic, were not ecologically significant until the Neoproterozoic Oxygenation Event, providing a rich source of organic matter for black shale formation [34,35]. Microbial respiration consumed dissolved oxygen in the water column and formed anoxic water in offshore silled basins and continental slope, which favored the development of black shales as well (Fig. 3).

### Earth's orbitally forced climate variation and sedimentary cycling

**Figure 4.**
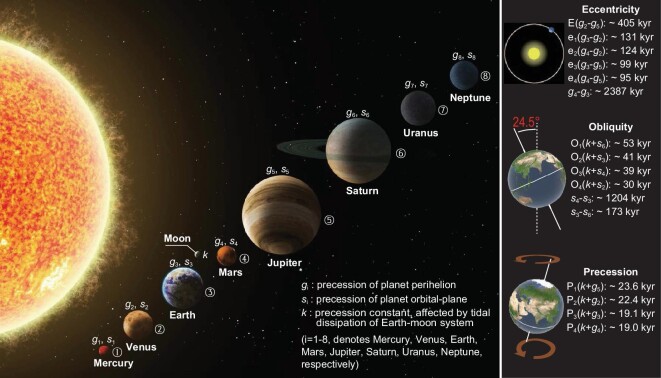
Planets of our solar system and major astronomical cycles of Earth. The parameters are from [38,39].

Solar radiation energy reaching Earth's surface shows periodic changes caused by variation in orbital parameters [36–39] (Fig. 4). In recent decades, the orbital cycle of black shale development has received close research attention and most of the work has been based on the Milankovitch theory. The Milankovitch theory was proposed in the 1930s [37] but was not widely applied until it was verified by robust geological evidence. Since 50 Ma, the main orbital parameters include long eccentricity (E: 2260 and 405 kyr), short eccentricity (e: 131, 124, 99 and 95 kyr), obliquity (O: 41 kyr dominant and 54, 39 and 29 kyr supplementary) and precession (P: 24, 22, 19 and 17 kyr) [38]. A representative example of orbitally forced changes in sedimentation is the clear depositional rhythm of limestone and mudstone in Cretaceous successions of the Italian Apennines, where the long and short eccentricity cycles of 405 and 100 kyr have been clearly identified by using high-resolution lithological and geochemical analyses [40]. During the Mesozoic Era, when black shales were frequently developed, orbital forcing was thought to have controlled fluctuations in sea and lake levels as well as pulses of anoxic events [41]. Moreover, during the Cretaceous warming period, wind strength and aridity in the provenance of sedimentary basins were markedly regulated by astronomical cycles, which in turn dominated the terrestrial hydrological cycle and nutrient transport [42]. Recent studies have found evidence of Milankovitch cycles in Mesoproterozoic to Neoproterozoic sediments. Of these records, the geochemical cycling of black shales and siliceous rocks of the Mesoproterozoic Xiamaling Formation was controlled mainly by precession [33], further supporting the close association between orbital forcings and the black shale depositions. However, the Precambrian obliquity and precession parameters differed significantly from those of the Phanerozoic owing to the influence of tidal dissipation and the variation of the Earth–Moon distance, which led to a faster Earth rotation and a shorter obliquity cycle compared with the present [39].

With the improvement in the dating accuracy of geological samples and the availability of various high-resolution records, geological evidence is emerging that cannot be explained by Milankovitch cycles. Some so-called ‘unconventional’ orbital amplitude modulation periods of >405 kyr have been found in the geological record, including 32–36, 26–27.5, 7–11, 2.4 and 1.2 Myr [43,44]. Although the impact of these unconventional cycles on insolation might be much smaller than that of the conventional cycles, they are widely recorded in sedimentary strata and can be strongly correlated with variations in organic carbon burial. The cycle of 26–36 Myr preserved in Mesozoic–Cenozoic sediments might be associated with astronomical cycles of the solar system, such as the solar system orbital cycle (26–28 Myr) [45] or the Nemesis gravitational cycle (26 Myr) [46]. The 2.4- and 1.2-Myr cycles have been widely reported from Phanerozoic strata and are mostly accompanied by weakening of the 405-kyr cycle. For examples, 1.2-Myr cycle enhancement and 405-kyr cycle weakening were recorded simultaneously in sediments deposited during the Early Triassic hothouse period [47]. In addition, astronomical calculations reveal a short obliquity period of 170 kyr, which is commonly accompanied by the 1.2-Myr and 40-kyr cycles and has a strong influence on climate in middle and high latitudes [38]. More importantly, this cycle has been identified in spectral analyses of the TOC data in sediments [48], suggesting its strong association with organic carbon enrichment.

**Figure 5.**
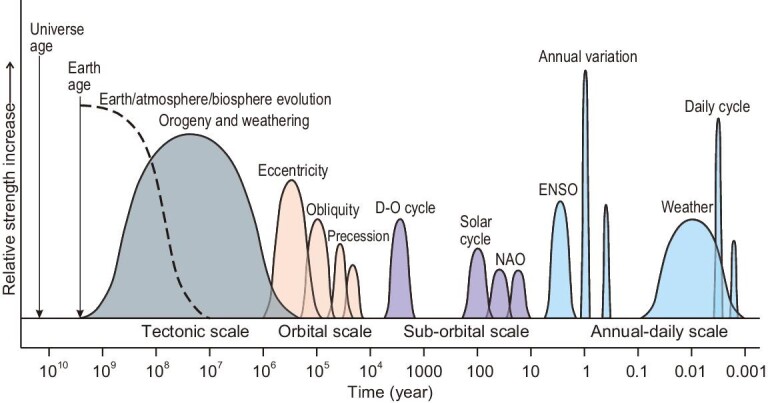
Climate change and sedimentary cycles on Earth, modified from [36]. ENSO, El Niño-Southern Oscillation; D-O, Dansgaard-Oeschger cycles; NAO, North Atlantic Oscillation.

In addition to the above conventional and unconventional astronomical orbital cycles, decadal to millennial solar activity cycles are considered as driving forcings for changes in Earth's climate on suborbital scales [36] (Fig. 5). These subscale cycles have been related to climate events since the late Quaternary ice age. The common solar activity cycles are the Schwabe cycle (11 yr), the Hale cycle (22 yr), the Gleissberg cycle (88 yr), the Sues or deVries cycle (195–235 yr), the longer-scale Eddy cycle (900–1080 yr) and the Hallstatt cycle (2400 yr) [38]. Of these, the 88-yr cycle may be the amplitude modulation of the 11-yr cycle, while the cycles of 500, 1000 and 2400 yr may be the modulations of the 88- and 200-yr cycles of amplitude variation in solar activity. Organic enrichments in the Upper Jurassic Smackover Limestone in the Gulf of Mexico show cyclicities of 0.5–3 and 5–15 cm, corresponding to the cycles of 8–48 and 80–240 yr, respectively, and are thought to be controlled by flood–drought climate change forced by sunspot activity [49]. Cyclicities of organic burial corresponding to cycles of 30–57, 81–110 and 360–500 yr have been identified in the Triassic Chang-7 black shale of the Ordos Basin [50], indicating that suborbital-scale climate change due to solar activity may be a key factor in determining nutrient transport fluxes, algal blooms and the formation of organic laminae.

**Figure 6.**
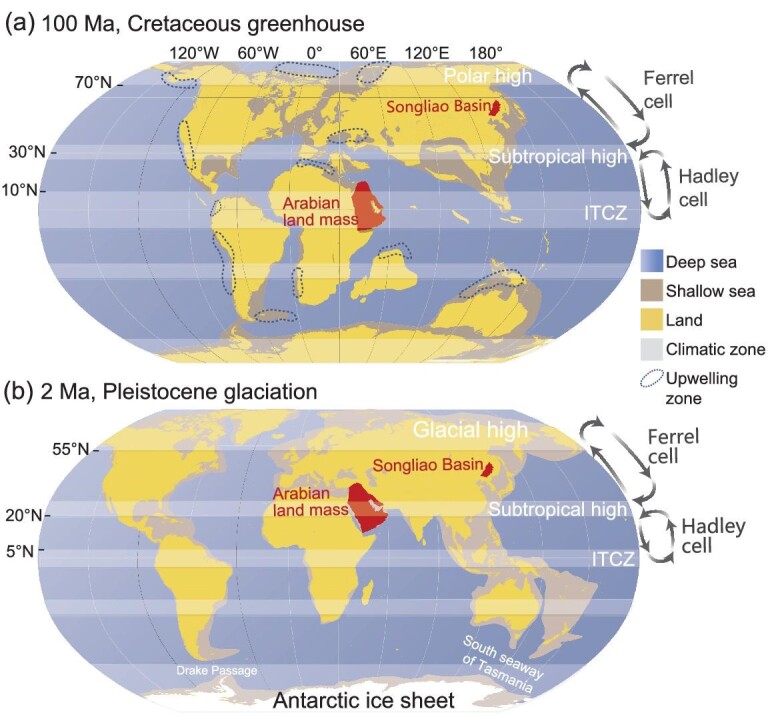
Climate zonation during the (a) Cretaceous greenhouse and (b) Pleistocene ice age, modified from [51,52]. ITCZ, Intertropical Convergence Zone.

Black shale represents material and energy transport and convergence. Oceanic and atmospheric circulation is the main mode of material and energy convergence and redistribution on Earth's surface. The Intertropical Convergence Zone (ITCZ) under the control of Hadley circulation at low latitudes is the most favorable location for the development of black shale due to the warm environments and high levels of primary productivity [33]. When landmasses drift into the ITCZ, organic-rich black shales can be developed over long intervals, providing the basis for the formation of hydrocarbon-bearing basins. In addition to the effect of Hadley circulation, the Ferrel circulation is the main control on climatic oscillations and organic carbon burial in the latitude range of 35°–65° [51], such as the Cretaceous Songliao Basin in northeastern China [51,52] (Fig. 6a). During the Cenozoic, the impact of Ferrel circulation on the development of terrestrial black shales in the northern hemisphere appears to quite remarkable, probably because the high-latitude continent-dominant settings are more sensitive to changes in weathering intensity than low or middle latitudes (Fig. 6b) [53].

### Weathering and transport of terrestrial nutrients

Black shales are composed predominantly of organic matter and clastic debris. The latter is derived from terrestrial weathering inputs. Owing to different climatic environments and geographic locations, the weathering half-life of LIPs or mountain regions can range from millions to tens of millions of years, thus allowing long-term supply of nutrients and detritus. During periods of frequent tectonic and volcanic activity, large volumes of greenhouse gases were released, generating warm periods and thereby enhancing the weathering and erosion of terrestrial materials, which could increase primary productivity and lead to the formation of black shales [24]. This tectonic–depositional process makes black shale highly related to climatic variation. The relationship between uplift, climate and weathering proposed by Raymo and Ruddiman [54] argues that tectonic uplift causes geomorphological instability, which, coupled with increasing precipitation, leads to increased surface erosion and enhanced chemical weathering. All of these conditions favor the development of black shales. Hence, global distributed black shales are often associated with large-scale orogenesis, which can be further linked to supercontinent reorganization. The level of primary productivity in modern oceans is positively related to the intensity of orogenesis. For example, the Himalayas and the Andes control surface weathering and primary productivity levels in the Ganges and Amazon Basins, respectively [55]. In contrast, during the orogenic quiescence of the mid-Proterozoic, black shales were relatively limited (Fig. 2), a phenomenon consistent with the first-order relationship between orogenesis, climate and weathering intensity.

On the other hand, large-scale orogenesis is often accompanied by the extrusion of marginal basins and the development of foreland basins in the Wilson cycle, when the accommodation space for black shale is limited. The oil and gas resources contributed by black shale in foreland basins are far less abundant than those in passive continental margins formed during supercontinent break-up. The latter account for 54.7% of global known oil and gas resources and >50% of the estimated oil and gas resources, as well as nearly 70% of the newly discovered global oil and gas reserves in 2012–16 [56,57]. After the formation of the Greenwell (1.2–1.1 Ga), Pan-African (0.6–0.5 Ga) and Hercynian (0.4–0.3 Ga) orogenic belts, the scale of black shale deposits and the number of oil and gas resources contained therein decreased markedly in each case. During the Phanerozoic, large amounts of black shale were deposited during the assembly and break-up phases of Pangea but, in between, the scale of black shale deposits decreased significantly [58]. Pulses of black shale formation and oxygenation events during the assembly of the supercontinent are likely to have been associated with the superior protection of sediments by continental crust, whereas black shales deposited during the break-up phase would have been prone to subduction together with descending oceanic slabs [59]. Accordingly, these differing processes may give the pseudo-impression that supercontinent assembly periods are more conducive to the development of black shale than break-up periods.

**Figure 7.**
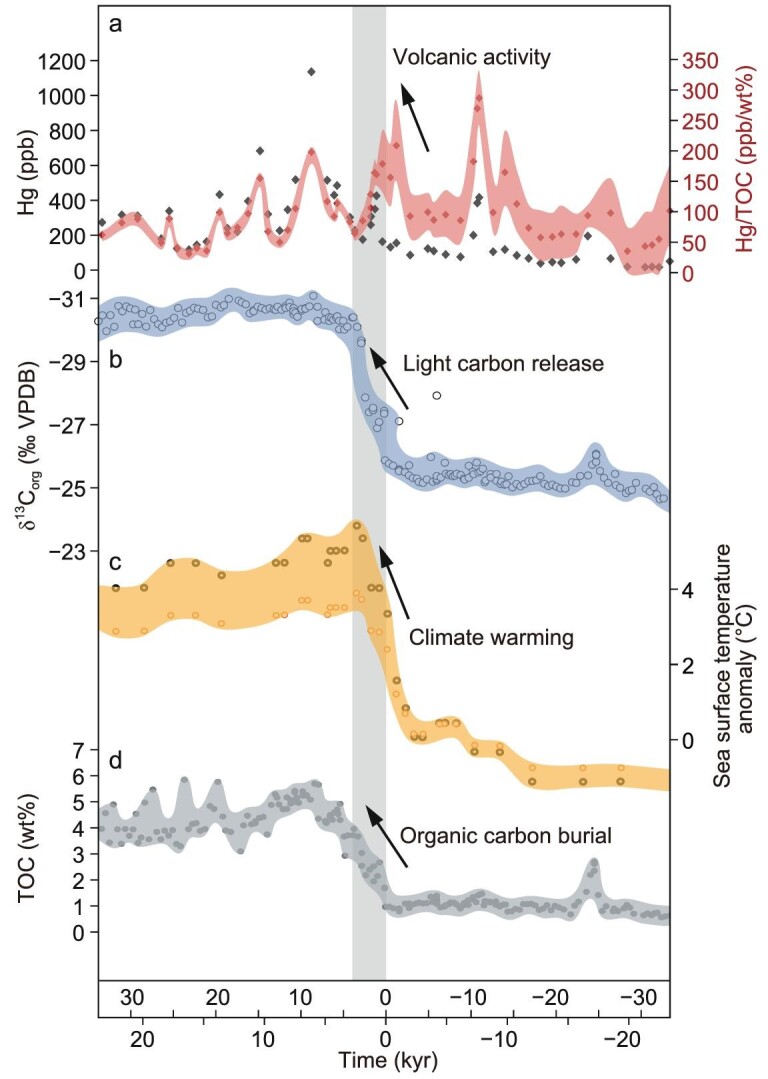
During the Paleocene Eocene Thermal Maximum, (a) volcanic activity (high Hg and high Hg/TOC values) and (b) light carbon isotope release (low δ^13^C values) led to (c) warming events and (d) increasing organic carbon burial (high TOC values). The data are cited from [63]. The different age scales are obtained by different core lengths and models. TOC, total organic carbon.

Compared with orogenesis, the duration of volcanic periods tends to be shorter with large uncertainties regarding their impact on black shale deposition. LIP events during supercontinent break-up could have enhanced terrestrial weathering by direct release of greenhouse gases or indirect release of thermogenic CO_2_ and CH_4_ through intrusions into carbonate or black shale deposits [60]. Freshly erupted basalt is very susceptible to chemical weathering [61], which works in a similar way to orogenesis in promoting the development of black shale. In accordance with the relationship between volcanic ash thickness and TOC values of black shales, it was proposed that medium-scale volcanic events are favorable for black shale formation, whereas small and large volcanic events are unfavorable [62]. However, this conclusion reflects the regional short-term impact of local volcanic events and does not seem to consider the long-term global effect of volcanic events. In addition, the following points should be noted. First, the thickness of volcanic ash does not necessarily reflect the size of the volcanic event. This is because volcanic ash thickness is strongly controlled by the transport efficiency and the distance from the volcanic vent to the sedimentary location. Second, many other factors that may influence the proposed relationship are not considered, such as the sedimentation rate, weathering intensity and primary productivity levels. Third, there is a significant time lag (thousands to tens of thousands of years) between volcanic eruption and black shale deposition, which represents the time span for weathering and erosion processes to transport nutrients into basins. For instance, during the Paleocene Eocene Thermal Maximum event, both climate warming and enhanced organic carbon burial occurred after volcanic activity and the large-scale release of ^12^C-enriched CO_2_ [63] (Fig. 7). Fourth, the importance of volcanic activity to black shale deposition could vary through geological time. As for the mid-Proterozoic oligotrophic oceans, volcanic activity should be a critical trigger for nutrient input. However, during the Phanerozoic Era, terrestrial and marine nutrients were already abundant. Therefore, volcanic activity was relatively less important and may even have produced negative feedback to marine nutrient availability via an active Fe cycle, thereby maintaining a relatively constant rate of organic carbon burial [64].

### Photosynthesis and organic matter degradation

The earliest life on Earth was probably chemoautotrophic archaea, followed by photosynthetic autotrophic prokaryotic bacteria (purple bacteria and cyanobacteria), with eukaryotes appearing last [29]. The composition of life during the pre-Ediacaran is still poorly understood, mainly because the fragility and microscopic nature of the organisms did not favor their preservation as fossils. In this context, lipid molecules from the organisms, also known as molecular fossils, are the most important tool for exploring and identifying early life. The parental source of sedimentary organic matter has been determined for various microbial populations, including cyanobacteria, green sulfur bacteria, purple sulfur bacteria, methanotrophic bacteria and various eukaryotic algae such as red algae, green algae and brown algae [34,35]. The organic carbon isotopes (δ^13^C_org_) of sedimentary organic matter vary markedly depending on the carbon source and carbon sequestration pathway [10,65]. Accordingly, the molecular fossils and organic carbon isotopic compositions are often utilized together to constrain the early evolution of life.

Cyanobacteria may be the oldest oxygenic photosynthetic organisms on Earth, with origins dating back to ∼2.7 Ga or even earlier [29], but organic matter in shales earlier than ∼2.5 Ga may have been derived primarily from chemoautotrophic or anoxygenic photosynthesis [65]. Cyanobacteria likely surpassed methanotrophs during the GOE and became the dominant primary producer and a major contributor to free oxygen [29]. Eukaryotic algae first evolved by the end of the Paleoproterozoic Era, with a relatively slow pace in biodiversification until the Neoproterozoic, when a rapid diversification occurred [34,35]. The general lack of eukaryotic records during the intervals in between has been debated. Strikingly, a recent discovery of abundant protosteroids, lanosterol or cycloartenol, from mid-Proterozoic sediments indicated that protosterol biota might have been widespread in aquatic environments at that time [35]. Fossils that can be confidently attributed to a known taxonomic clade of crown group eukaryotes include *Bangiomorpha* and *Proterocladus*, both dating back to ∼1 Ga [66,67]. The great radiation of algae during the Cryogenian laid the foundation for the Ediacaran emergence, the Cambrian explosion and the Ordovician radiation of metazoans. It is suggested that ∼90% of marine and lake biomass during the Phanerozoic was microplankton, including cyanobacteria, acritarchs and algae. In particular, cyanobacteria and chemoautotrophic archaea could have still flourished in oceanic anoxic environments that are often featured by the extinction of heterotrophs, providing organic matter for black shales. Results of experimental cultures also confirm that aliphatic biopolymers present in cyanobacteria can contribute to the formation of kerogen via selective preservation [68]. Moreover, the highly aliphatic, recalcitrant algaenan isolated from two cyanobacteria, *Chlorella* and *Sphaerellopsis*, are similar in structure to the algaenan of oil-bearing algae and have a hydrocarbon potential of >50% [69]. During the Cretaceous Oceanic Anoxic Event (OAE) 1b, black shales deposited on both sides of the North Atlantic contained abundant isoprenoid tetraether membrane lipids, isoprenoid alkanes in a free state or bound to macromolecules and ether lipid compounds such as bi/tricyclic dipentane tetraethers, all of which indicate a significant contribution of archaea to sedimentary organic matter [70]. The δ^13^C_org_ compositions of monomeric biomarkers and kerogen revealed that ≤80% of the TOC during the OAE 1b was derived from chemoautotrophic marine non-thermophilic archaea, rather than from eukaryotic algae as conventionally thought [71]. Therefore, the contribution of prokaryotic bacteria and archaea to organic matter and the hydrocarbon potential of Phanerozoic black shales is non-negligible.

**Figure 8.**
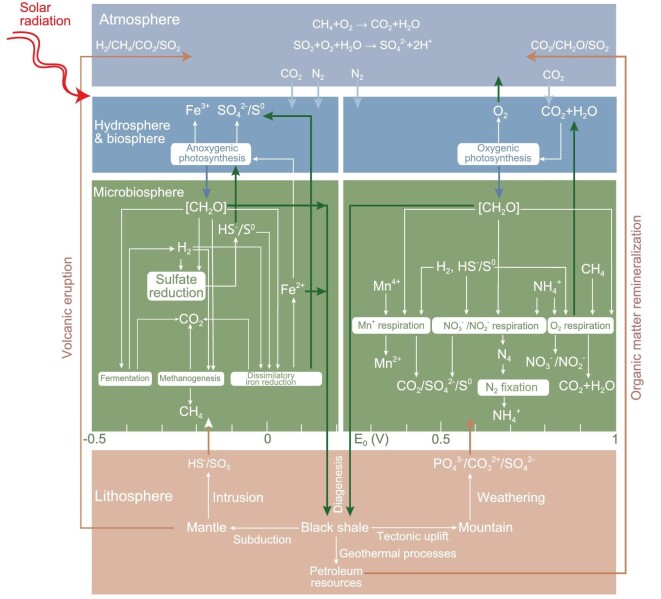
A generalized model showing the cycling of carbon and related elements in the Earth system, modified from [72].

Organic matter produced by photosynthetic organisms in the surface layer of oceans or lakes undergoes complex degradation by heterotrophic microbial activity through the water column and during diagenesis. According to the thermodynamics of the processes and the energy available to the organisms, the sequence of the microbial degradation of organic matter is aerobic respiration, denitrification, dissimilatory manganese reduction, dissimilatory iron reduction, microbial sulfate reduction and methanogenesis [10]. The anaerobic sequence can substantially destroy evidence of surface water ecosystems or obscure primary information with that of deep anaerobic microorganisms. The cycling of organic matter by microbial respiration can change the form and enrichment degree of key elements, which may further affect redox states of the water column and even the atmosphere [72] (Fig. 8). Microbial sulfate reduction might have been the most important redox reaction in Phanerozoic oceans, generating sulfite intermediates and toxic H_2_S, which inhibit the degradation and consumption of organic matter by aerobic microorganisms and methanogenic bacteria, providing a favorable anaerobic environment for the preservation of organic matter [10]. In Precambrian oceans, sulfate input could have bound Fe^2+^ through H_2_S generated by microbial sulfate reduction. During this process, the generated FeS (eventually converted into FeS_2_) enters the sediment, converting the Fe–C cycle to the Fe–S–C cycle, and inhibits the degree of organic matter degradation by iron reduction, which in turn facilitates organic matter preservation [10,73]. The prevalence of anoxic waters also favors the long-term preservation of DOC. DOC could have constituted a huge carbon reservoir during the mid-Proterozoic and may have been several times larger than the amount of buried organic carbon in black shales [10]. This huge DOC reservoir likely persisted until the Ediacaran, when it began to fade with increasing oceanic oxygenation, but it may have increased again during anoxic events of the Phanerozoic oceans [9]. Indeed, even in the modern oceans, of which 95% are oxic, the DOC reservoir has been estimated as 680 Gt, which is comparable to the amount of atmospheric CO_2_ (720 Gt) [8]. The presence of large DOC reservoirs makes organic carbon burial a dynamic process with unbalanced carbon input and sink, which may partially explain the relative scarcity of black shales through the Precambrian time.

The importance of microbial respiration to marine and lacustrine ecosystems also involves the release of organic-carbon-bound nutrients (e.g. phosphorus, nitrogen and iron) to sustain primary production [1,74]. Phosphorus and nitrogen are regarded as the most important nutrients limiting primary productivity and are bound together with carbon at a certain ratio (C:N:P = 106:16:1, the Redfield ratio) in organic matter [74]. The ‘Fe hypothesis’, which is based on the significant negative correlation between Fe and CO_2_ concentrations in Antarctic Vostok ice cores, suggests that Fe has a prominent fertilizing effect on phytoplankton in high-nutrient, low-chlorophyll seas, promoting photosynthesis and the uptake of atmospheric CO_2_ [75]. More detailed studies showed that the Fe fertilization effect also differs substantially between the warm and cold periods of Earth, with the effect being more pronounced during cold periods, corresponding to the scarcity of terrestrial nutrient inputs [76].

It is worth noting that the degree of organic matter decomposition is dependent on a number of factors, including settling velocity, water depth, oxidant availability and sedimentation rate. Among them, the impact of the sedimentation rate has been long debated and regarded as a double-edged sword on organic carbon preservation [11,77]. Elevated sedimentation rates can reduce the exposure time of organic matter to oxygen, thereby minimizing the influence of aerobic respiration. However, such a scenario may dilute TOC as well. For examples, in deltaic environments where sedimentation rates can be extraordinarily high [78], the TOC contents are typically <1 wt% [77]. The Mesoproterozoic Xiamaling shales characterized by slow depositional rates and a quiescent tectonic background exhibit TOC of ≤20 wt% [10], supporting the enrichment of organic matter by condensed sedimentation.

## BLACK SHALE FORMATION AND THE EVOLUTION OF EARTH’S HABITABILITY

Black shale is a record of biological activity and an archive of Earth evolution. Advances in studies of the Earth system have given insights into how Earth's layers interact to supply and redistribute materials and energy, as discussed in detail below. Organisms play crucial roles in producing and mineralizing organic matter, generating chemical byproducts, gradually changing the surface settings and climate, producing a variety of mineral resources and promoting the habitability of Earth.

### Tracking Earth's oxygenation history

An oxygen-bearing atmosphere is one of the key features of Earth's habitability. The main source of free oxygen on Earth is biological photosynthesis. While releasing oxygen, photosynthesis generates organic matter, which is subsequently buried to form black shale. Hence, black shale is a reliable geological tracer of Earth evolution. In recent years, redox-sensitive elements (e.g. Mo, U and Cr) and their isotopes in black shales have played an important role in the investigation of Earth's oxygenation. These metallic elements can show significant enrichment and isotopic fractionation in black shales, which are considered to be closely related to the redox state of the atmosphere and ocean. Studies of U isotopes in Archean and Paleoproterozoic shales constrained the origin of oxygen-producing photosynthesis into two possible periods: 3.0–2.9 and 2.7–2.5 Ga [79]. However, the extent of oceanic oxygenation was likely small and in the form of oxygen oasis, which was not sufficient to allow free oxygen to accumulate in the atmosphere. The pronounced isotopic fractionations of Mo and U in black shales during the GOE indicated a substantial increase in the areas of oxygenated seafloor [23,80]. During the Mesoproterozoic Era, elemental and isotopic evidence in black shales supports the existence of episodic oxygenation in the oceans, with atmospheric oxygen levels reaching 1% or likely ≤4% of the present-day levels [81,82]. A Mesoproterozoic Oxygenation Event is therefore proposed (Fig. 2) [14]. The relatively elevated oxygen concentrations could have sustained effective terrestrial weathering and vigorous cycling of redox-sensitive elements.

Earth's oxygenation process was also active during the late Neoproterozoic and Paleozoic Eras. After the Marinoan glaciation, the abundances of trace metals and prominent isotopic offsets indicate extensive oxygenation of Earth's surface environments. In particular, during the Shuram carbon isotope excursion, the degree of marine oxygenation might have been close to modern levels inferred by using U-isotope mass balance modeling [83]. Another important oxygenation event occurred during the early Cambrian (520 Ma), when Mo and U isotopes of the Niutitang Formation in South China recorded strong fractionations and high organic carbon burial fluxes in the ocean [84]. During the Precambrian and early Paleozoic, brief episodes of oceanic oxygenation were commonly accompanied by black shale formation, but atmospheric oxygen levels fluctuated. After the Devonian Plant Explosion, atmospheric oxygen levels gradually stabilized at modern concentrations, as indicated by sedimentary and geochemical evidence [85].

### Genesis of hydrocarbon and metal–mineral resources

Organic matter in black shales can generate hydrocarbon after being geologically or artificially heated. Hydrocarbon molecules expelled from black shales could have accumulated in high-porosity rocks and then form oil and gas reservoirs under seal conditions. The discovery of authigenic pores in black shale indicated that large volumes of shale oil and gas can be trapped in the source rocks [56]. Methanogenesis by microorganisms during the early burial of organic matter can also form large bio-gas fields and gas hydrates in submarine and permafrost areas, which constitute clean gas resources [86]. Hence, the spatial and economic boundaries of oil and gas exploration are being expanded by these unconventional hydrocarbon resources.

Black shales are also enriched in many rare metals and precious elements. These elements are referred to as critical minerals or key metals because most of them are essential for modern society but their supply is at high risk [87]. As a large number of key metals are redox-sensitive, they are easily oxidized during surface processes, subsequently precipitated in reducing waters and then enriched in black shales. For instance, the lower Cambrian Niutitang Formation, found in certain regions of South China, is rich in trace metals, such as nickel, molybdenum, uranium, rhenium and platinum group elements [88]. In addition, during the subduction of sediments into the mantle, the accumulated metals can be activated and enriched to form key mineral deposits, such as porphyry copper and porphyry molybdenum [59,89]. These processes demonstrate the critical role of the evolution of Earth's surface environment and its interaction with the lithosphere in the formation of key metal deposits.

### Prediction of climate change

Terrestrial and marine ecosystems represent important sinks of CO_2_ through photosynthesis and are referred to as ‘ecosystem carbon sinks’. The global organic carbon burial flux is ∼0.17 Gt yr^−1^ [1], offsetting ∼3% of the increase in atmospheric CO_2_ concentrations. The sedimentary burial flux of organic carbon is not uniformly distributed on Earth's surface, but is highly focused in nearshore environments along continental margins. Marine sediments obtained from ocean drilling programs and water-column traps have revealed that organic matter deposition in the ocean is not continuous, but is instead characterized by episodic enrichments [90,91]. However, the exact ways in which organic carbon burial and black shale development are related to climate change still need to be assessed. For example, during the massive burial of organic carbon in the Permian Junggar Basin, microbial methanogenesis of organic matter released large amounts of CH_4_, a potent greenhouse gas [92], which may have contributed to the demise of the late Paleozoic ice age and the initiation of global warming [93]. A study on the Middle Miocene showed that heterotrophic bacterial metabolism and organic matter degradation in the oceans were accelerated by increased temperature, resulting in a decrease in the rate of organic carbon burial on the seafloor [94]. These investigations point to a complex relationship between organic carbon burial and climate change.

## MAJOR SCIENTIFIC QUESTIONS AND FUTURE DIRECTIONS REGARDING BLACK SHALE

Our exploration of Earth, much like our comprehension of black shale, began with a focus on individual factors before progressing to the study of interactions across multiple spheres. A more integrated understanding of black shale, grounded in multi-sphere interactions and Earth System Science, will enhance our grasp of the evolution of Earth's habitability. Furthermore, it will guide us to a more sustainable utilization of our resources on Earth.

### Black shale research from an Earth System Science perspective

Research into black shale has provided geological evidence for the evolution of Earth's surface environment, but the temporal resolution and reliability of the relevant data still need to be enhanced. Further progress in our comprehension of black shale can be achieved by embracing an Earth System Science perspective and undertaking more multidimensional research investigations. In terms of observation, we should not only study biology and mineralogy at the micron or even nanometer scale to understand their contributions to the material and energy cycles, but also study the lithosphere, hydrosphere and biosphere at a scale of ≥10 km to understand the processes and mechanisms of the interactions among multiple spheres. In terms of data, high-precision and coupled analyses of element concentrations and isotopic ratios are required. We should also establish high-resolution (1–10 kyr) geological, geophysical and geochemical databases to obtain accurate information about Earth’s evolution. In addition, the mechanisms of orbitally forced and solar-radiation-driven surface climate change, cyclic sedimentary deposition and organic matter enrichment are not yet well known, especially the reconciliation of multiscale cycles and the differential response of different latitude zones.

Taking the Xiamaling Formation as an example, although substantial advances have been achieved regarding the depositional mechanism of organic-rich shales, further exploration under the framework of Earth System Science is still warranted. The relative contributions of volcanism and hydrothermal activity require a more nuanced evaluation. Questions remain as to the role of continental weathering in transporting nutrients, as well as the process of organic matter degradation through diverse biogeochemical reactions.

### Exploration and development of hydrocarbon and metal–mineral resources

The development of a country depends on guaranteed resources. The 2022 edition of the *World and China Energy Outlook* notes that oil and gas together account for >50% of global primary energy consumption and that this proportion will remain similar until 2035. Major economies around the world have understood and grasped the security of petroleum supply as a major strategic issue. As the gradual depletion and deterioration of conventional hydrocarbon resources in medium-depth and shallow strata occur, the exploration of deep and ultra-deep oil and gas, shale oil and gas, and deep-seawater hydrocarbon, with black shale as the main target, will be essential for guaranteeing energy security. In recent years, deep-seawater hydrocarbon has become a major component of global resources. However, the formation mechanism and distribution of organic-rich shale in many deep-seawater areas are poorly understood, thereby restricting larger-scale exploration for deep-seawater resources. In addition, in 2021, 21 strategic resources in China were dependent on imports and 12 strategic resources had a dependence of >70%, including chromium, manganese, nickel, cobalt, uranium, platinum group elements and other elements that are abundant in black shales. Many unanswered scientific questions remain concerning the enrichment of low-abundance key metals, especially the biogeochemical process of the co-enrichment of organic matter and key metals in black shale, the sources of key metals, the influence of sedimentation processes on the enrichment and the mineralization of organic carbon. Opportunities in these areas range from geological testing to experimental simulations, which will shed new light on our understanding of organic and inorganic mineralization.

### Carbon sequestration for mitigating climate change

In recent years, climate change, characterized by rising temperatures and increasing variability, has been considered to have affected the habitability of Earth, with the main cause being the dramatic increase in the concentration of greenhouse gases since the Industrial Revolution. As climate change is now recognized as a major issue for the future development of humanity, the world's major economies have announced ‘carbon peaking and carbon neutrality’ goals, with the core objective of trying to mitigate anthropogenic CO_2_ emissions and global warming. In September 2020, the Chinese government announced its double carbon targets of achieving peak carbon output by 2030 and carbon neutrality by 2060. Carbon emissions reduction and carbon sequestration are the two most important aspects for achieving the goal of carbon neutrality. Accordingly, it will be necessary to further strengthen research into the effectiveness and mechanisms of carbon sequestration, such as ocean–land carbon storage and the microbial carbon pump, and to better utilize modern marine, lake, forest and grassland resources for carbon storage. Ecological carbon sequestration can be achieved by increasing the reservoir sizes of sedimentary organic carbon, authigenic carbonate rocks and recalcitrant DOC. In addition, geological storage after CO_2_ capture is also regarded as an effective method for mitigating climate change. However, there are many technical challenges involved in such storage, as well as risks posed by gas leakage and as-yet unforeseen types of geological hazard. Thus, it is necessary to conduct thorough investigations of the safety of CO_2_ geological storage. Predictions and solutions for the future will likely depend on knowledge and solutions from the past, and understanding Earth evolution with respect to black shale deposition should provide important information for such solutions.

In conclusion, the organic carbon cycle and the development of black shale have been controlled by multi-sphere interactions within the Earth system, which have led to the evolution of a habitable Earth and the provision of geological resources for humans, such as hydrocarbons and metallic minerals. The extensive use of fossil energy and resultant release of CO_2_ emissions have influenced Earth's climate and now pose a threat to the future habitable environment for humanity. Given the clarion call of habitability, scientists need to better understand the organic carbon cycle and how it influences climate and resources through interdisciplinary research. Technical breakthroughs are needed for geological carbon storage and to realize ecological carbon sequestration with the aim of forming a new framework of carbon cycle to achieve efficient resource discovery, rational energy utilization and environmental sustainability.
